# Gait scoring in dogs with thoracolumbar spinal cord injuries when walking on a treadmill

**DOI:** 10.1186/1746-6148-10-58

**Published:** 2014-03-05

**Authors:** Natasha J Olby, Ji-Hey Lim, Kellett Babb, Kathleen Bach, Cullen Domaracki, Kim Williams, Emily Griffith, Tonya Harris, Audrey Muguet-Chanoit

**Affiliations:** 1 College of Veterinary Medicine, North Carolina State University, Raleigh, North Carolina, USA; 2 Member of the Center for Comparative Medicine and Translational Research, Raleigh, North Carolina, USA

**Keywords:** Coordination, Footstep sequence, Paresis, Paralysis, Regularity index, Intervertebral disc herniation, Spinal cord injury

## Abstract

**Background:**

An inexpensive method of generating continuous data on hind limb function in dogs with spinal cord injury is needed to facilitate multicentre clinical trials. This study aimed to define normal fore limb, hind limb coordination in dogs walking on a treadmill and then to determine whether reliable data could be generated on the frequency of hind limb stepping and the frequency of coordinated stepping in dogs with a wide range of severities of thoracolumbar spinal cord injury.

**Results:**

Sixty-nine neurologically normal dogs of different body sizes including seven lame dogs were videotaped walking on the treadmill without prior training and all used the lateral gait of right fore, left hind, left fore, right hind (RF-LH-LF-RH). Severely paraparetic dogs were able to walk on the treadmill for a minimum of 75 seconds, scoring of which generated data representative of function in animals with extremely variable gaits. Fifty consecutive stepping cycles were scored by three observers in 18 dogs with a wide range of disability due to acute thoracolumbar spinal cord injury using a stepping score (hind limb steps/fore limb steps ×100), and a coordination score (coordinated hind limb steps/total hind limb steps ×100). Dogs were also scored using a previously validated ordinal open field score (OFS). Inter- and intraobserver agreement was high as assessed with Cronbach’s alpha test for internal reliability. The stepping and coordination scores were significantly correlated to each other and to the OFS.

**Conclusions:**

Dogs with naturally occurring spinal cord injury can walk on a treadmill without prior training and their hind limb function can be scored reliably using a stepping score and coordination score. The only requirements for data acquisition are a treadmill and appropriately positioned video camera and so the system can be used in multicentre clinical trials to generate continuous data on neurologic recovery in dogs.

## Background

Acute spinal cord injuries are common in dogs, in large part due to the high prevalence of explosive Hansen type 1 intervertebral disc herniations in chondrodystrophoid breeds such as the dachshund [1]. The large number of otherwise healthy young dogs that suffer these injuries has led to increasing interest in performing placebo controlled, blinded clinical trials [2-4] both to improve current therapies for dogs, and to identify promising therapies that will translate to human medicine [5,6]. In order to perform a clinical trial, it is vital that outcome can be quantified accurately, and, in the case of multicentre trials, that the method of data collection is accessible to multiple centres. In experimental studies, outcome is often quantified by use of several different measurements that assess different parameters. For example, the widely used BBB ordinal scale provides a measure of open field walking [7], but this is often complemented with measurements of stride length and foot step sequences [8,9] and ability to perform a task such as beam walking or negotiating a grid [8]. Current outcome measures used in dogs largely rely on ordinal scales that score unassisted movement in the open field. The scale used most commonly in canine neurology is the modified Tarlov scale [10], that scores gait somewhat crudely, although reliably, from 0 to 5. However, with only 5 grades, this scale lacks discrimination. Several researchers have supplemented this scale with ordinal assessments of proprioceptive placing and nociception [2,11], but still lack the ability to subdivide the recovery of dogs with incomplete ambulatory skill. A more discriminative ordinal scale has been developed to aid in the quantification of dogs’ recovery [12] and has been used prospectively to generate data for study design [13], but the data remains ordinal rather than continuous. The use of tasks that require specific skills and therefore training prior to injury are less appropriate for a clinical caseload that cannot be trained ahead of time. More objective data on the spatiotemporal gait characteristics can be generated using a pressure sensitive walkway (PSW) [14] and kinematic analysis [15,16] but this requires specialized and expensive instrumentation.

There is a need for an inexpensive method of generating continuous data that accurately quantifies recovery from spinal cord injury in dogs, to complement the ordinal scales already in use. A system that quantifies stepping ability and coordination would allow assessment of the function of multiple spinal cord tracts. The aim of this study was to describe the quadrupedal coordination of normal dogs walking on a treadmill and to use this data to develop a treadmill based gait scoring system for dogs with a wide range of severity of thoracolumbar spinal cord injuries to accurately and reliably quantify their hind limb function.

## Methods

### Normal step sequence in dogs walking on a treadmill

**Figure 1:**
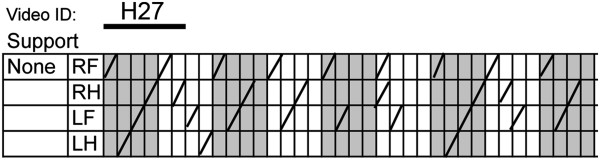
**An example of a scoring sheet.** A footfall is documented with a line in the appropriate box. Scoring is started with the right fore limb and each block of 4 columns represents one step cycle with two forelimb steps per cycle.

Healthy dogs were recruited from the staff and students of the North Carolina State University College of Veterinary Medicine and underwent a neurological and orthopaedic examination including evaluation of their gait for lameness, paresis or ataxia. Dogs with neurological deficits were excluded, but dogs with orthopaedic abnormalities were included in order to determine whether coincident orthopaedic disease altered step sequence as many neurologic patients may have coincident orthopaedic conditions. Dogs were walked on a treadmill (Ironman Inspire) at three different walking speeds to determine whether this influenced coordination. First the treadmill speed was adjusted to achieve the pace that appeared most comfortable to the dog (defined as natural walk). A video camera was positioned to capture movement of all 4 limbs and at least one minute of continuous walking was videotaped. The treadmill speed was then reduced to the slowest speed that would keep the dog walking (slow walk) and the videotaping repeated, and then it was increased to the point just below the threshold of producing a trot (fast walk), and the videotaping was repeated. The videotapes were then reviewed using Windows Movie Maker to view them frame-by-frame, and the sequence of footsteps was recorded using a chart (Figure 1). One observer scored a total of 25 step cycles (100 steps) for each dog.

### Development of stepping and coordination scores

Dogs with a wide range of different severities of thoracolumbar spinal cord injury were videotaped on the treadmill without any support of the pelvis, when their tail base was held, and finally, when walking with sling support under their abdomen which was adjusted to allow them to step even when unable to bear their body weight. Treats were used to keep the dogs engaged and walking; no training was performed prior to data collection in order to determine whether this test could be applied to untrained pet dogs. All dogs were taking part in clinical trials and all animal use was approved by the NCSU Institutional Animal Use and Care Committee (protocol numbers: 09-068-O and 11-009-O). The presence and sequence of steps in all 4 limbs were scored using a scoring chart (Figure 1). When the dogs were unsupported, a hind limb step was defined as protraction of the hind limb to a point level with or cranial to the coxofemoral joint with progression to foot placement (the ventral or dorsal surface of the paw making contact with the treadmill) followed by weight bearing of the hind quarters sufficient to raise and support the pelvis during the stride (from foot placement to the foot leaving the ground again). If the dorsal aspect of the paw was placed on the ground this was noted with a D in place of a line in the score sheet for additional analysis of paw placement if desired. If the dog was being supported either by holding the base of their tail or by sling, weight bearing was not assessed; rather, any step that went through the whole sequence of protraction, foot placement and stride as defined above were counted. For the purposes of step sequence, the timing of the step was documented at the point at which the foot left the ground. Complete stepping sequences involving all 4 limbs in the correct sequence (as based on data developed during assessment of normal dogs) were counted and the total number of fore and hind limb steps were counted. Two scores were calculated from the treadmill data. The ‘stepping score’ is the ratio of hind limb to fore limb steps. The ‘coordination score’ is the ratio of coordinated hind limb steps to the total number of hind limb steps.

Severely paraparetic dogs with a general proprioceptive ataxia can have extremely variable gait, going from dragging their hind limbs, to stepping and back to dragging. In order to determine how many footsteps should be scored in order to accurately represent an individual’s ambulatory ability, two dogs with severe paraparesis and extremely variable gaits were videotaped for as long as they could comfortably tolerate walking on the treadmill at a natural speed. The number of pelvic limb steps taken was then quantified for the entire videotape, and then for 15, 45 and 75 seconds evenly split into the first, middle and last 5, 15 and 25 seconds of the videotape respectively. The stepping scores from the three different partially scored tapes were correlated with the scores from the complete tape and the r^2^ value was recorded. Paired t-tests were performed to look at the pairwise differences in scores between the full video and the clips.

### Inter- and intraobserver reliability and comparison with ordinal scales

Four different observers independently scored treadmill videotapes from 18 dogs with a range of different gait severities; each dog was scored by three observers. In addition, videotapes from five of these dogs (with a range of disability) were scored three times by one of the observers with a minimum of two weeks between observations. Videotape of each dog walking in the open field was also scored by a previously described ordinal scale [12] (Table 1). Inter- and intraobserver agreement for stepping, coordination and open field scores was assessed using the Cronbach’s alpha test for internal consistency. The relationship between the open field and treadmill generated scores was examined using Pearson’s correlation coefficient. All statistical evaluation was performed using jmp10 pro (SAS Institute, Raleigh, NC). P values <0.05 were taken as significant.

**Table 1:** **Ordinal scale used to score gait**[12]

**Score**	**Description**
0	Paraplegic, no nociception.
1	Paraplegic plus nociception.
2	Paraplegic with voluntary tail wag.
3	Minimal non-weight bearing protraction of pelvic limb (movement of 1 joint)
4	Non-weight bearing protraction of pelvic limb limb with > 1 joint involved < 50% of time.
5	Non-weight bearing protraction of pelvic limb limb with > 1 joint involved > 50% of time.
6	Weight bearing protraction of pelvic limb limb < 10% of time.
7	Weight bearing protraction of pelvic limb limb 10-50% of time.
8	Weight bearing protraction of pelvic limb limb >50% of time.
9	Weight bearing protraction 100% of time with reduced strength of pelvic limbs. Mistakes >90% of time (crossing of pelvic limbs, scuffing foot on protraction, standing on dorsum of foot, falling).
10	Weight bearing protraction 100% of time with reduced strength of pelvic limbs. Mistakes 50-90% of time.
11	Weight bearing protraction 100% of time with reduced strength of pelvic limbs. Mistakes <50% of time.
12	Ataxic pelvic limb gait with normal strength but mistakes > 50% of time (lack of coordination with thoracic limb, crossing of pelvic limbs, skipping steps, bunny hopping, scuffing foot on protraction, standing on dorsum of foot).
13	Ataxic pelvic limb gait with normal strength but mistakes < 50% of time.
14	Normal pelvic limb gait.

## Results

### Normal step sequence

Sixty two neurologically and orthopedically normal dogs ranging from toy to giant breeds were evaluated. Breeds represented included 25 mid to large sized mixed breeds, 4 small mixed breeds (<10 kg), 5 Labrador Retrievers, 5 Golden Retrievers, 2 Airedale Terriers, 2 Bearded Collies, 2 Bouvier des Flandres, 2 Jack Russell Terriers, 2 Boston Terriers and 1 each of the following: Boxer, German Shepherd Dog, Rhodesian Ridgeback, Giant Schnauzer, Staffordshire Bull Terrier, Borzoi, Greyhound, Great Dane, Beagle, Corgi, Australian Shepherd, Miniature Poodle and a Basset Hound. Seven lame dogs (2 Golden Retrievers, 2 Greyhounds, 2 large mixed breed dogs and a pitbull terrier) were also assessed. These dogs had degenerative joint disease (DJD) affecting the elbows (n = 1), hock (n = 2) and the coxofemoral joints due to hip dysplasia (n = 4). In all dogs, their DJD was producing mild lameness. All dogs (sound and lame) adapted rapidly to the treadmill generating data suitable for scoring. Slow walking speeds ranged from 0.6 to 1.2 miles per hour (mph) (mean 1.02 mph), natural walking speeds ranged from 1 to 1.8 mph (mean 1.44 mph), and fast walking speeds ranged from 1.6 to 2.6 mph (mean 1.86 mph). All dogs walked with the standard stepping sequence of right fore (RF), left hind (LH), left fore (LF), right hind (RH), also known as a lateral walk [17], at the natural and fast walking paces. At the slow speed, 8 dogs adopted a lateral walk the majority of the time, but tended to falter and use random patterns for a few paces. Three of these dogs were lame. The lateral stepping pattern was adopted as normal coordination for scoring of dogs with spinal cord injuries.

### Stepping and coordination scores

**Figure 2:**
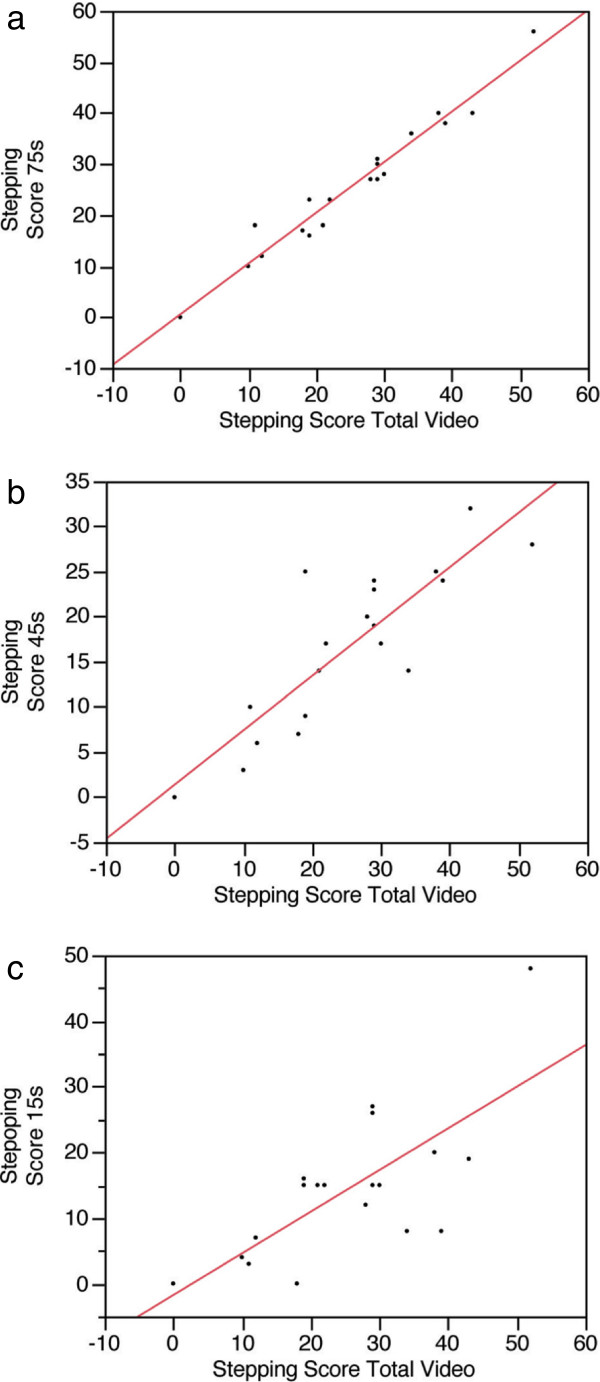
**Correlation of the stepping score for the entire videotape of a treadmill session with scoring of 75 (a), 45 (b) and 15 (c) second excerpts of the videotapes.** The r^2^ value for **(a)** is 0.96, for **(b)** is 0.733 and for **(c)** is 0.52.

A total of 20 dogs with thoracolumbar spinal cord injuries (18 due to acute intervertebral disc herniations and 2 due to traumatic injury) were used in this part of the study. The stepping scores for 19 different videotapes from two dogs with extremely variable gait due to severe spinal cord injuries (both dogs were >6 months post injury) were calculated from the entire videotape (mean duration: 187.4 s; range 77 to 304 s) and from the first, middle and last 5 seconds (total of 15 seconds or approximately 20 fore limb steps or 10 potential step cycles), 15 seconds (total of 45 seconds, approximately 70 forelimb steps or 35 potential step cycles) and 25 seconds (total of 75 seconds, approximately 100 forelimb steps or 50 potential step cycles) (Table 2). There was no significant difference in stepping score between the 75 second clip and the total video, but there was a significant difference in score between the 15 and 45 second clips and the total video (Table 3 and Figure 2)*.* Scoring of 50 potential step cycles was therefore adopted as the standard scoring protocol.

**Table 2:** Stepping scores when scoring the entire videotape, and then the first, middle and last 5, 15 and 25 seconds of videotapes of paraparetic dogs walking on the treadmill

**Videotape ID**	**Total video length (s)**	**Stepping score total video**	**Stepping score 75 s**	**Stepping score 45 s**	**Stepping score 15 s**
D1-1	77	10	10	3	4
D1-2	245	21	18	14	15
D1-3	256	28	27	20	12
D1-4	140	29	30	24	27
D1-5	209	12	12	6	7
D1-6	301	29	27	19	26
D1-7	122	22	23	17	15
D1-8	185	29	31	23	15
D1-9	304	38	40	25	20
D2-1	95	19	16	9	15
D2-2	100	19	23	25	16
D2-3	195	11	18	10	3
D2-4	79	0	0	0	0
D2-5	198	52	56	28	48
D2-6	258	30	28	17	15
D2-7	228	43	40	32	19
D2-8	282	39	38	24	8
D2-9	201	34	36	14	8
D2-10	113	18	17	7	0

**Table 3:** Comparison of the stepping score generated by scoring the entire video tape versus 75, 45 or 15 seconds of the videotape of dogs walking on a treadmill using paired t tests

**Comparison**	**Mean difference**	**t-statistic**	**p-value**
Total vs. 75 seconds	−0.37	−0.60	0.56
Total vs. 45 seconds	8.74	5.54	<0.001*
Total vs. 15 seconds	11.05	5.31	<0.001*

*denotes a significant p value.

Videotapes of 18 dogs that had suffered thoracolumbar spinal cord injuries were scored by three observers. Ten of these dogs had suffered severe spinal cord injuries resulting in chronic (> 6 months post injury) loss of nociception and non-ambulatory status. Eight dogs had suffered less severe spinal cord injuries and were scored at 4 weeks following their injury at which time they were recovering strong motor function with varying amounts of ataxia. This allowed the scoring technique to be applied to dogs with a wide range of disability. Dogs quickly adjusted to the treadmill and videotape suitable for scoring was obtained in them all. Non-ambulatory dogs were not videotaped for a prolonged period without support; enough videotape was obtained to be sure that there was no weight bearing motor function, thus avoiding abrasion of the skin of their ventrum and medial thighs on the treadmill. They were then videotaped with support to allow scoring of all non-weight bearing stepping movements.

All observers scored dogs from the same time point in the videotape, identified as the first time point from which 50 step cycles could be scored. The mean scores for each dog are provided in Table 4. Inter-observer reliability was calculated for all the dogs as a single group and then for the chronically paralyzed and recovering dogs separately, to assess the ability to score consistently at different levels of function and in different patient populations. Scores between observers were highly correlated with Cronbach’s alpha measure of internal consistency approaching a value of 1 (Table 5). Likewise, intraobserver reliability was extremely high (Table 5). Using Pearson’s correlation coefficient, all scores were highly correlated, with the strongest relationship identified between the OFS and the stepping score (Table 6).

**Table 4:** Scores for paraparetic dogs (values are the mean from all three observers)

**Dog**	**Stepping score**	**Coordination score**	**OFS**
1	5.33	0	3
2	0	0	0
3	22	18.1	4
4	0	0	0
5	37.33	17.97	5.33
6	18.33	11.2	4
7	16	0	5
8	45.67	33.61	5
9	44.33	24.73	5
10	43.67	28.37	5
11	89.24	57.53	9
12	66.07	5.5	9
13	97.73	89.83	13
14	80.93	40.43	11
15	46.75	47.9	7.33
16	69.1	29.47	8
17	101.33	59.13	10.33
18	99	85.9	12.33

Dogs 1-10 were non-ambulatory and their treadmill scores were generated with tail support. Dogs 5 and 6 had acute traumatic injuries, while the rest of the dogs had acute intervertebral disc herniations. OFS: open field score [12].

**Table 5:** Reliability between three independent observers (interobserver) and one observer scoring three times (intraobserver) using three different scores of hind limb function

**Group scored**	**Stepping score correlation (Cronbach’s alpha)**	**Coordination score correlation (Cronbach’s alpha)**	**OFS correlation (Cronbach’s alpha)**
Interobserver: chronic non-ambulatory dogs (n = 10)	0.992	0.991	0.997
Interobserver: recovering ambulatory paraparetic dogs (n = 8)	0.991	0.991	0.978
Interobserver: whole group (n = 18)	0.998	0.992	0.997
Intraobserver: (n = 5)	0.997	0.994	1

The dogs were considered as a whole group, but also separated into chronic non-ambulatory and recovering ambulatory paraparetic dogs because the quality of the gait of these two groups of dog is quite different.

**Table 6:** Correlation between the treadmill based stepping, coordination and open field scores using Pearson’s correlation coefficient

**Group evaluated**	**Variable 1**	**Variable 2**	**Correlation**	**P value**
Chronic non-ambulatory dogs (n = 10)	Stepping	Coordination	0.9405*	<0.0001
	Stepping	Open field	0.8279*	0.0031
	Coordination	Open field	0.6684*	0.0346
Recovering ambulatory paraparetic dogs (n=8)	Stepping	Coordination	0.6692	0.0695
	Stepping	Open field	0.8050*	0.0159
	Coordination	Open field	0.7069*	0.0499
Whole group (n=18)	Stepping	Coordination	0.8657*	<0.0001
	Stepping	Open field	0.9504*	<0.0001
	Coordination	Open field	0.8362*	<0.0001

All correlations denoted with * are significant.

## Discussion

The treadmill based scoring system presented here represents an inexpensive method of quantifying gait reliably in spinal cord injured paraparetic dogs with a wide range of disability. We demonstrated that neurologically normal, orthopedically sound and lame dogs of different sizes walk with a lateral gait (RF-LH-LF-RH) at a natural speed on the treadmill and we developed a scoring sheet that allows documentation of foot fall sequence from videotapes of dogs walking on the treadmill. Scores of both coordination and stepping frequency were developed and found to have high inter- and intraobserver reliability and to correlate closely with a previously developed ordinal scale of open field walking.

Our major requirements of a gait scoring system in spinal cord injured dogs were that it did not use expensive or specialized equipment, to allow multiple centres to acquire data for clinical trials; that it could be applied to dogs of varying size and conformation, over a wide range of disability; that it would not be affected by existing orthopaedic disease; that it did not require prior training; and that it provided discriminating continuous data with a high level of repeatability. Ordinal open field gait scoring systems are practical and have demonstrated utility and reliability [2,7,11,12,18], but do not generate continuous data and are limited in sensitivity. Such scoring systems are usually complemented by the addition of scoring techniques that assess different aspects of gait and provide continuous and highly discriminative data when used experimentally (e.g. [8,19]). The spatiotemporal characteristics of gait are altered dramatically by spinal cord injury and can be quantified using kinematic analysis systems and PSWs. However, a major challenge posed by attempting to quantify this type of data is the inherent variability in many of the stride characteristics due to differences in size and conformation between breeds [14-16]. Accounting for covariates such as tibial length and velocity of walking can allay this problem and data generated in dogs with spinal cord disease using the PSW demonstrated that the fore limb stride length, swing and stance time shortens, and the hind limb swing time increases significantly with spinal cord injury [14]. However, correlation to injury severity was lacking, likely because only independently ambulatory dogs could negotiate the walking surface. Moreover, the need to perform multivariate regression to factor in covariates complicates analysis in a clinical caseload of patients. Detailed kinematic analysis underlined the presence of inter-individual variability in many parameters, and indeed, the most consistent measures generated quantified the variability in both diagonal coupling time (time elapsed between fore limb and contralateral hind limb placement) and lateral paw placement [15,16]. In normal dogs, these parameters do not vary, resulting in values close to zero. Changes in these parameters are sensitive markers of spinal cord injury severity [15,16] and such data collection is objective but unfortunately costly. With the constraints of cost and availability, the use of kinematic data collection systems and PSWs was not viable for our purposes.

Given the need for a low cost system, we adapted strategies used in experimental models of spinal cord injury to count footsteps and evaluate step sequences. The two systems used most commonly in rodent work are foot print analysis accomplished by inking feet and evaluating the foot steps left on a paper surface [8], and catwalk analysis, a system in which the subject walks on a side-illuminated glass sheet that illuminates each paw as it is placed on the glass [20]. The subject is videotaped from below and the videotapes analysed after the fact. These systems also allow collection of data such as base of support, stride length, and foot position [8,9], data that we could not collect from our system, but data that is problematic due to the covariates encountered in dogs. We chose to collect our data when dogs were walking on a treadmill in order to allow us to control walking speed for individual dogs throughout testing [15,16]. It is reported that people walk differently on a treadmill versus the open field [21], and so data on coordination in normal dogs walking on a treadmill was collected at the start of the study. The use of support for non-ambulatory dogs enables the investigator to quantify limb movements in even the most severely affected animals and has been shown not to affect gait patterns in normal dogs [15]. By focusing simply on quantification of the numbers of steps taken and the sequence of footfalls, the issues associated with quantification of factors such as stride length were avoided.

Descriptions of quadrupedal gait date back to the 1800s when Muybridge [22] used photography to document gait sequences in a range of species walking in the open field. At that time he described a canine walking gait as RF, LH, LF, RH (now called the lateral gait) and included documentation of a pacing gait (in which both fore and hind limbs on the same side are placed on the ground simultaneously) as dogs increased speed to transition to a trot. Beyond this early work, detailed literature on the frequency with which particular stepping sequences are used in walking dogs on and off the treadmill is limited [17]. Footfall sequences have been described in detail in rats walking in the open field and a total of six different sequences, composed of two mirror versions of three basic patterns: cruciate, alternating and rotary, were documented [23]. Of these patterns, 80% of female wistar rats used the ‘alternating b’ pattern that corresponds to the lateral pattern in dogs [19]. Our data revealed that a large cohort dogs of different sizes, and including lame dogs, adopted the lateral pattern [17,22] when walking at different speeds on a treadmill. While some of these dogs did occasionally adopt a pacing gait when walking at faster speeds in the open field (unpublished data), this was not recorded while they were walking on the treadmill. A small number of dogs had brief periods of more random stepping sequences when walking at the slow speed, in particular the lame dogs, likely because of attempts to shift weight off the lame limb(s), but all walked with the lateral pattern when the speed of the treadmill was increased. We therefore adopted the lateral pattern as our definition of normal foot sequences when scoring videotapes of gait.

We were interested in both the number of hind limb steps that are taken and the fore limb hind limb coordination because it is proposed that animals can recover walking ability through increased activation of the local central pattern generators and through recovery of ascending and descending input via the long tracts of the spinal cord [24]. The sequence of foot falls in quadrupedal mammals is controlled by both local ascending and descending propriospinal pathways that influence the thoracic and pelvic limb central pattern generators, as well as by supraspinal input [25,26]. The coordination of fore and hind limbs is therefore affected by spinal cord injuries [15,27]. It has been shown that accurate quantification of coordination between fore and hind limbs provides a sensitive measure of spinal cord injury in rats [28] that complements ordinal scales such as the BBB scale. In addition, the recovery, or lack thereof, of coordination in the presence of recovery of stepping ability potentially provides information on the mechanism of motor recovery.

The parameters that we scored were the ability to take a hind limb step, and the percentage of hind limb steps that occurred within a normal stepping cycle with the rationale of providing a simple measure of stepping frequency and strength (the stepping score) and of ascending and descending input (the coordination score). An alternative method of computing coordination known as the regularity index (RI) has been reported in rats [28] and can be calculated readily from our data by multiplying the number of footsteps taken in a normal stepping sequence by 4 to give the total number of coordinated steps and dividing by the total number of steps taken [28]. The stepping score can be used to assess motor function by quantifying the number of weight-bearing steps, related to frequency and strength of stepping, and by quantifying the number of steps taken when the dog is walked with support. The extremely high correlation between the stepping score and the OFS supports the view that the stepping score quantifies degree of successful ambulation. Recovery of coordination is less complete than stepping, as reflected in the lower coordination scores. The coordination score correlated less closely (although still significantly) with the OFS than the stepping score, reflecting the fact that the OFS, unlike the BBB scale used in rodent spinal cord injury research [7], does not focus on quantifying coordination between fore and hind limbs. It is interesting to note that recovery of coordination was quite variable in the ambulatory dogs, for example, dogs 17 and 18 were both stepping with their hind limbs as frequently as their forelimbs, but in dog 17 only 60% of the hind limb steps were coordinated in a normal stepping cycle, compared with 86% in dog 18.

The definition of a footstep used was refined during the development of the scoring system to enhance scoring consistency and to capture all stepping movements. The observers in this study had very different backgrounds and included a veterinary student with no prior training in gait analysis, and three veterinarians one of whom had never worked in the field of spinal cord injury and two who were experienced in this field. The high repeatability of the data between these individuals and upon repeat scoring by one individual supports the utility of this scoring system. However, wider use of the system will allow more rigorous assessment of the extent of training needed to maintain consistent results in a wider population of observers. The major drawback of this scoring system is that, while videotapes of dogs can be obtained very efficiently, it is time intensive to score the videotapes. Time taken to score each videotape was not recorded by the observers in this study, but typically, it takes approximately 5 minutes to score a neurologically normal dog or a completely paraplegic dog, while paraparetic dogs could take as long as 30 minutes to score due to the need to watch the videotapes at reduced speed to accurately identify each foot step.

## Conclusions

In conclusion, normal dogs with and without orthopaedic disease walk in a lateral walking pattern at a natural walking speed on the treadmill. The hind limb gait deficits in spinal cord injured dogs can be quantified reliably using a simple treadmill-based system that generates continuous data on both coordination and stepping ability. There is an extremely high correlation between the established OFS and the stepping score, both of which quantify the number of steps taken, albeit in different ways. The coordination score generates information on the recovery of a normal stepping cycle, providing more detailed information on the reestablishment of communication between fore and hind limbs.

## Abbreviations

RF-LH-LF-RH: Right fore, left hind, left fore, right hind; OFS: Open field score; PSW: Pressure sensitive walkway; mph: Miles per hour.

## Competing interests

The authors declare that they have no competing interests.

## Authors’ contributions

NJO designed the experiments, analysed data and wrote the manuscript; JHL, KeB, CD and AMC collected and analysed data; KaB, KW, TH collected data, EG performed statistical data analysis. All authors read and approved the final manuscript.
